# Supplementary material for: Effect of booster vaccination against Delta and Omicron SARS-CoV-2 variants in Iceland
Source: Nat Commun. 2022 Sep 28;13:5701. doi: 10.1038/s41467-022-33076-4 (PMC9517986; doi:10.1038/s41467-022-33076-4)
Supplement: Supplementary file 1 — Supplementary Information [file 41467_2022_33076_MOESM1_ESM.pdf]

Supplementary Appendix

Table of contents

Supplementary methods 2
- Sequencing 2
- RNA extraction 2
- Reverse transcription and PCR amplification 2
- Sample preparation for ONT Sequencing 2
- ONT Sequencing 2
- Analysis of sequence data 3
- SARS-CoV-2 testing 3
- Statistical analysis 3

Supplementary figures 5
- Figure S1 5
- Figure S2 6
- Figure S3 7
- Figure S4 8
- Figure S5 9
- Figure S6 10
- Figure S7. 11
- Figure S8. 12
- Figure S9. 13

Supplementary tables 14
- Table S1 14
- Table S2 14

Supplementary references 21

Supplementary methods

Sequencing

The viral genomes of all positive PCR samples were sequenced at deCODE genetics, the methods have been previously described[1] and are reproduced here for the benefit of the reader. Reverse transcription and multiplex amplicon PCR were performed on the basis of information provided by the Artic Network initiative (https://artic.network/) to generate complementary DNA and sequencing libraries. Samples were sequenced using either Illumina or ONT sequencing technologies. Illumina sequencing was performed using MiSeq sequencers (MiSeq v2 reagent kits) with 2 x 150 cycle paired-end reads, with up to 48 multiplexed samples per run. Samples for ONT were multiplexed using native barcodes and sequenced using either GridION or PromethION flowcells, version R.9.4.1. Details are provided in the Supplementary methods.

RNA extraction

Viral RNA samples were extracted from swabs (96 samples per 40 min run) using the Chemagic Viral RNA kit on the Chemagic360 instrument from Perkin Elmer, with 300/100 µL input/output volume(s), respectively. Each step in the workflow was monitored using an in-house LIMS (VirLab) with 2D barcoding (Greiner, 300 µL tubes) of all extracted positive samples.

Reverse transcription and PCR amplification

Reverse transcription (RT) and multiplex PCR was performed based on information provided by the Artic Network initiative (https://artic.network/) to generate cDNA. In short, extracted viral RNA was pre-incubated at 65 °C for 5 min in the presence of random hexamers (2.5 µM) and dNTP´s (500 µM). Sample cooling on ice was then followed by RT using SuperScript IV (ThermoFisher) in the presence of DTT (5 mM) and RNaseOUT inhibitor (Thermo Fisher) for 10 min at 42°C, followed by 10 min at 70 °C. Multiplex PCR of the resulting SARS-CoV-2 cDNA was performed using a tiling scheme of primers, designed to generate overlapping amplicons of approximately 400 bp. Two PCR reactions were performed for each sample using primer pools 1 and 2, respectively (Table S7). PCR amplification was done using the Q5® Hot Start High-Fidelity polymerase (New England Biolabs) with primers at 1 µM concentration. The reactions were performed in an MJR thermal cycler with a heated lid at 105 °C, using 35 cycles of denaturation (15 sec at 98 °C) and annealing/extension (5 min at 65 °C). The resulting PCR amplicon pools A and B were combined, purified using 1X Ampure XP magnetic beads (Beckman Coulter) and quantified using the Qubit dsDNA high-sensitivity assay kit (Thermo Fisher).

Sample preparation for ONT Sequencing

Pooled amplicons (50-400 ng) were prepared for ONT sequencing using native barcoding with up to 24 barcodes. The first step in the procedure was end-repair using NEBNext Ultra II End Prep Reaction Buffer and Enzyme mix, respectively (NEB/E7546L). Samples (50 µL) were incubated in an MJR thermocycler at 20 °C for 10 min, followed by 10 min at 65 °C and cooling at 4 °C. Following AMPure XP clean-up (1X), the native barcodes (NBD104/NBD114) were ligated by mixing 20 µL purified sample, 2.5 µL barcode and 25 uL blunt/T4 ligase mastermix (NEB/M0367L). The mixture was incubated at room temperature on a rotator mixer for 10 min. Samples were purified by AMPure XP and quantified using the Qubit dsDNA high-sensitivity assay kit. Samples were pooled appropriately based on yield and/or Ct values from previous RT-qPCR measurements, with up to 24 samples/pool. Pooled DNA samples (up to 90 uL) were next incubated on a rotator mixer with 5 uL adapter mix II (AMII/ONT), 20 uL NEBNext Quick ligation reaction buffer and 10 uL Quick T4 DNA ligase (NEB/E6056L) for 10 min at room temperature followed by AMPure clean-up and bead washing with 2x120 µL of short fragment buffer (SFB/ONT). Final pooled sample libraries were resuspended in 35 uL of EB buffer and used immediately for sequencing.

ONT Sequencing

Pooled libraries were sequenced on either GridION X5 or PromethION sequencers, using the MinION (R9.4.1/FLO-MIN106D) and PromethION (R9.4.1/FLO-PRO002) flowcells, respectively. Following priming of the flowcells, the libraries were mixed with sequencing buffer (SQB) and loading beads (LB) and loaded onto the flowcells in the appropriate volume depending on flowcell type (75 µL or 150 µL). Sequencing runs were performed using on-board, high-accuracy basecalling (Guppy) with generation of FASTQ files for each barcode. Data acquisition varied from 1-4 hrs depending on the number of samples/pool and run performance. At least 100K reads were acquired per sample (barcode).

Analysis of sequence data
We aligned amplicon sequences to the reference genome of the SARS-CoV-2 (GenBank number, NC_045512.2). For Illumina sequences we used the latest Burrows–Wheeler Aligner (BWA-MEM) and variants were called with sequencing utilities bcftools as previously described.[2] For ONT sequences, sequence alignment and variant calling was performed using the Artic Network pipeline (https://artic.network/) with default parameters.

SARS-CoV-2 testing
To assess the variant of PCR positive samples where sequencing was not yet available, we used SGTF (Spike Gene Target Failure) property of the omicron variant (B.1.1.529) on the TaqPath™ COVID-19 CE-IVD RT-PCR Kit (Catalog number: A48067, ThermoFisher), otherwise the analysis remained unchanged[2].

Statistical analysis

The full linear model used for antibody concentration was

$$log(antibodyconcentration) \sim vaccine + covariates$$

and was fitted using the glm function in R with a Gaussian family. For the vaccine, the vaccine administered was taken as a factor with BNT16b2- BNT16b2 as a reference. For covariates, sex was regressed as a factor and age as a continuous variable in years. Confidence intervals for estimates of parameters were computed using the confint function in R. Since the 95% confidence interval is computed on a log-scale, the reported confidence interval was projected to the original scale using the exponential function. For age we report the confidence interval of the parameter per 10 years to aid the reader, this is done by multiplying the confidence interval by 10 before taking the exponent.

For inhibition levels we used the following model

$$log1p(inhibition\ levels) \sim vaccine + \ covariates$$

where $log1p(x) = log(1 + x)$. All covariates were treated as with the model for antibody concentration. Confidence intervals were computed the same way, but projected using the inverse of log1p, $exp(x) - 1$.

To estimate the parameters of variables affecting relative risk we used as Cox proportional hazard model with the following model

$$relative\ risk \sim vaccine + \ sex + pspline(age) + d_2 + d_3$$

which was fitted using the `coxph` function in the R library `survival`. Here d_2 is the number of months following the second vaccination before the start of the study, similarly for d_3. For the first time period, no persons had recived a booster dose and the d_3 was not included in the model. To account for vaccinations that occured within the study window we used time varying covariates as implemented by the `tmerge` function where the covariate was delayed by 14 days after vaccination. Persons who were diagnosed with covid during the study period exited the study at the date of diagnosis.

The adjusted survival curves were computed using the fitted model and the *conditional* method to compute survival curves using the `ggsurvplot` function in the `survminer` R library.

Supplementary figures

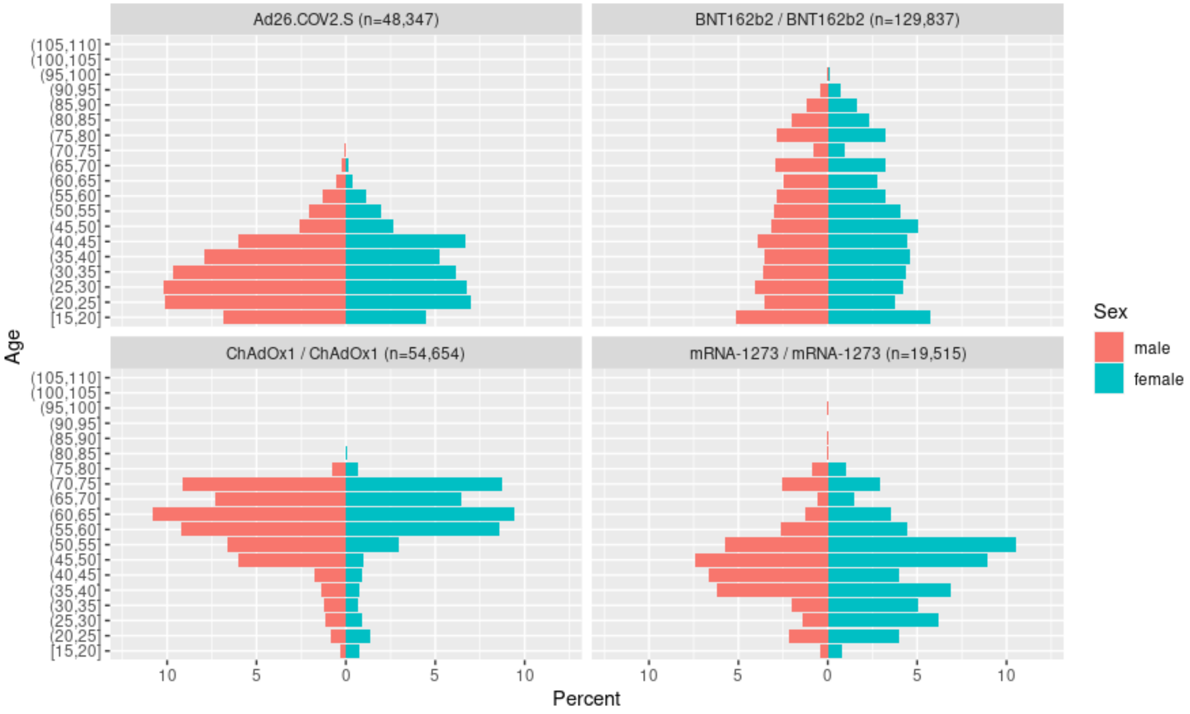

Figure S1 Age and sex distribution of primary schedule vaccination of the four vaccines deployed in Iceland by November 30th 2021.

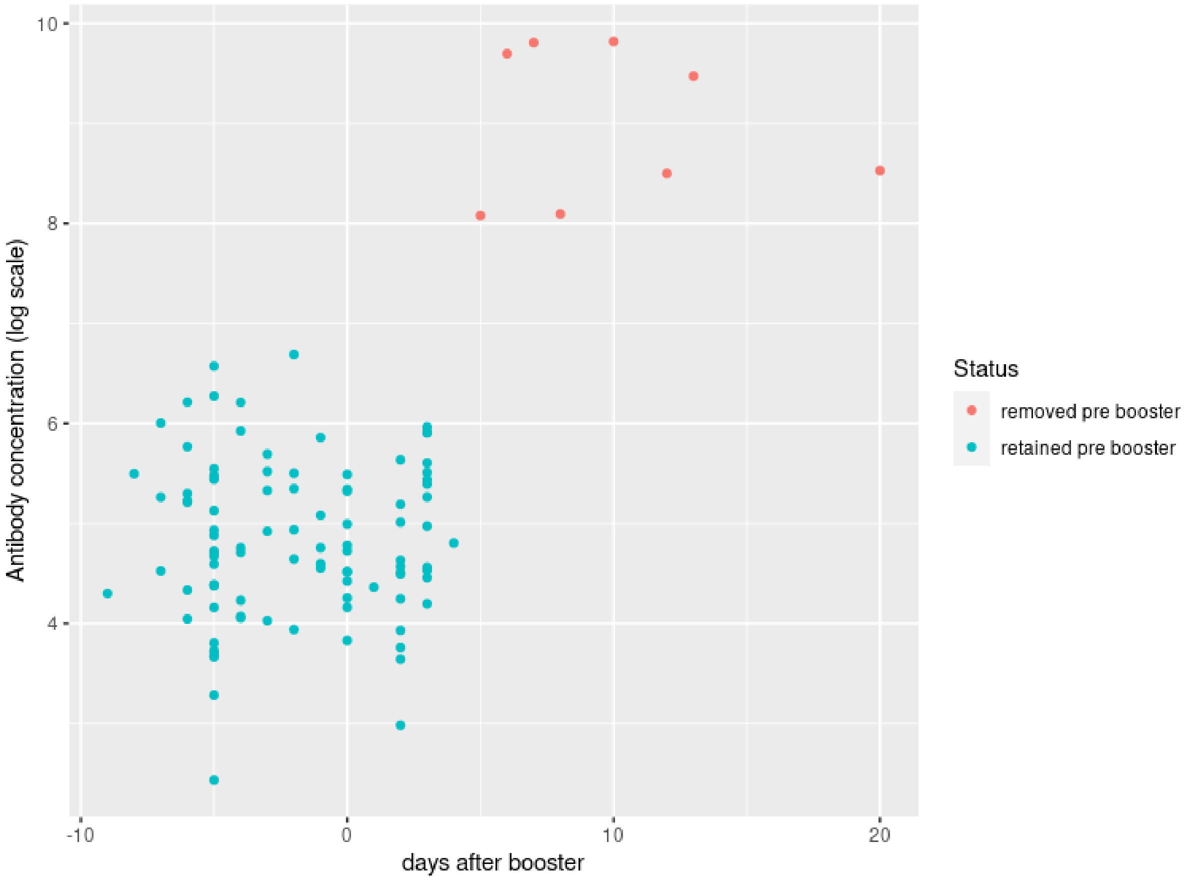

Figure S2 Anti-Spike-RBD antibody levels by number of days before or after booster dose. Red dots show samples removed from further analysis. Eight persons (red) gave their first sample 5 days or more after the mRNA booster and their antibody levels were considerably higher than in the pre-booster group (65x, 95% CI: 45-155x, $p<2x10^{-16}$). However, two months after the booster their antibody levels had lowered and were indistinguishable from the other samples in the post booster group (95% CI: 0.7-2.1x, p=0.44). The effect size and p-values were estimated using two-sided t-test, not correcting for multiple comparisons.

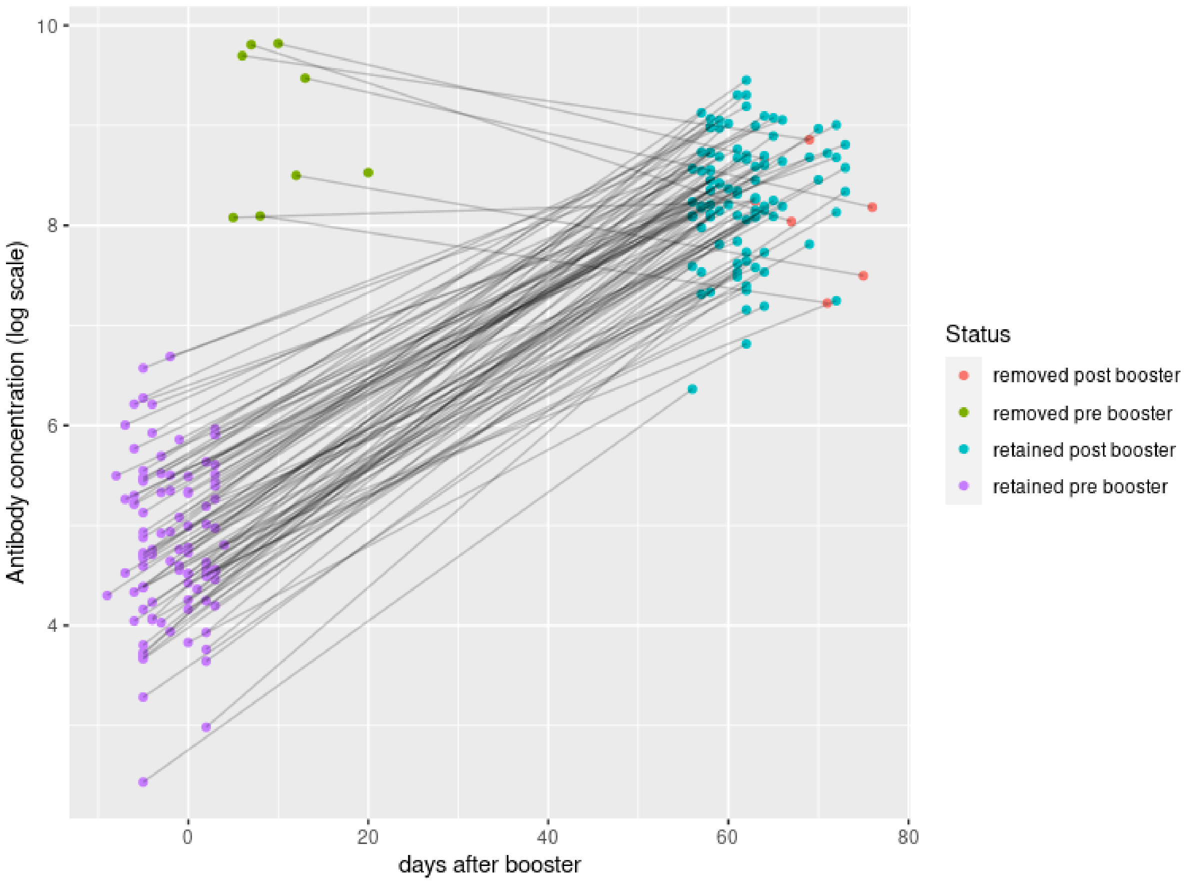

Figure S3 Anti-Spike-RBD antibody levels by number of days before or after booster dose in all persons with two samples.

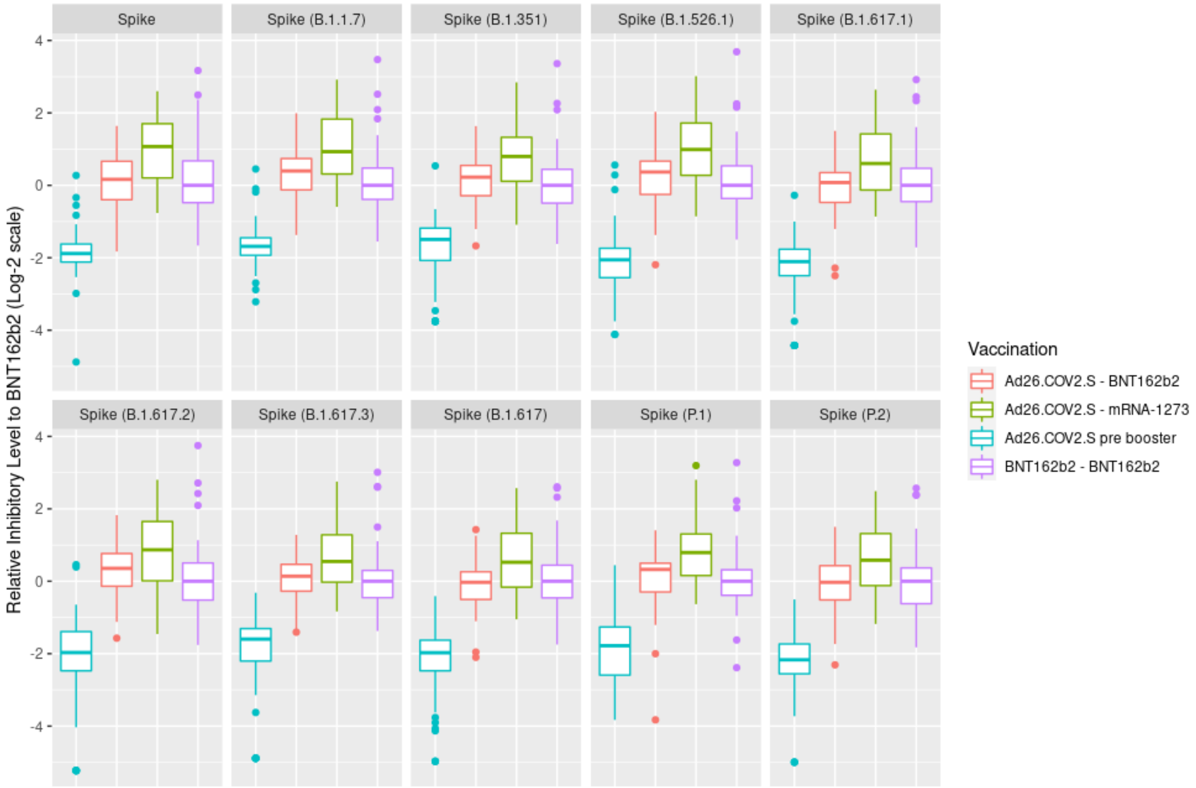

Figure S4 ACE2-Spike binding inhibition levels across Spike protein variants in persons vaccinated with Ad26.COV2.S before and after receiving mRNA booster vaccination. Data normalized to the two dose BNT162b2 reference group. We measured levels of ACE2-Spike binding inhibition in serum using nine additional Spike variants, including delta. The ACE2-Spike binding inhibition levels prior to booster were lower in recipients of Ad26.COV2.S than two dose BNT162b2 for all Spike variants tested, as observed for the original Spike (Table S1). Similarly, the Ad26.COV2.S-mRNA-1273 group showed the highest relative ACE2-Spike binding inhibition levels post-booster, whereas Ad26.COV2.S-BNT162b2 did not show a statistically significant difference from the two-dose BNT162b2 mRNA reference group, irrespective of Spike variants. Ad26.COV2.S-BNT162b2 (n=33), Ad26.COV2.S-mRNA-1273 (n=66), Ad26.COV2.S-pre booster (n=99), BNT162b2-BNT162b2 (n=59). Each boxplot shows that 25th and 75th percentile values (boxes) with the median as a center line, the whiskers depict 1.5 times the inter-quartile range and individual outlier values are shown as points.

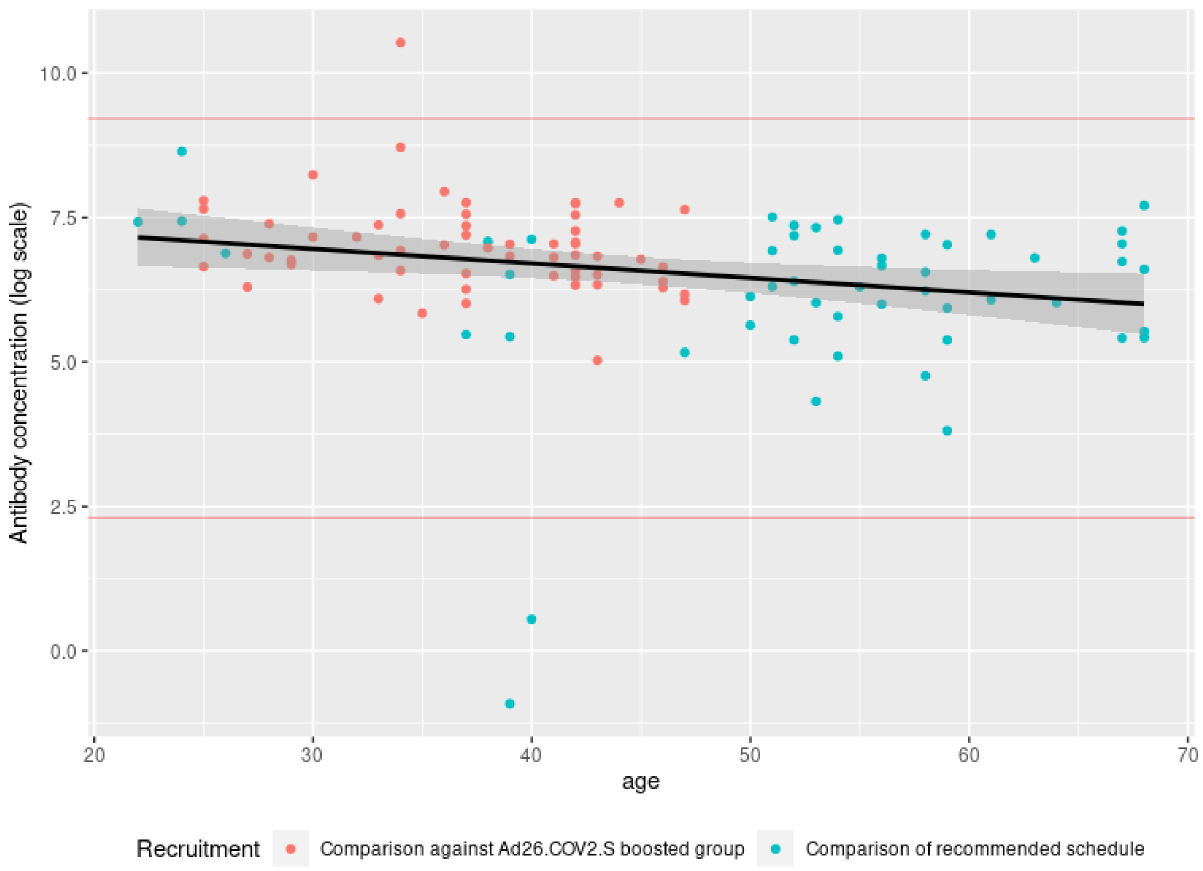

Figure S5 Anti-Spike-RBD antibody levels two dose BNT162b2 vaccinees: the reference group for Ad26.COV2.S receiving mRNA booster (red, N=52)(Figure 2B), reference group for the recommended two dose schedules (cyan, N=59)(Figure 2A). Red lines show the limits of outliers removed at 10 and 10000 U/ml. Shaded are represents the 95% confidence interval of the linear fit.

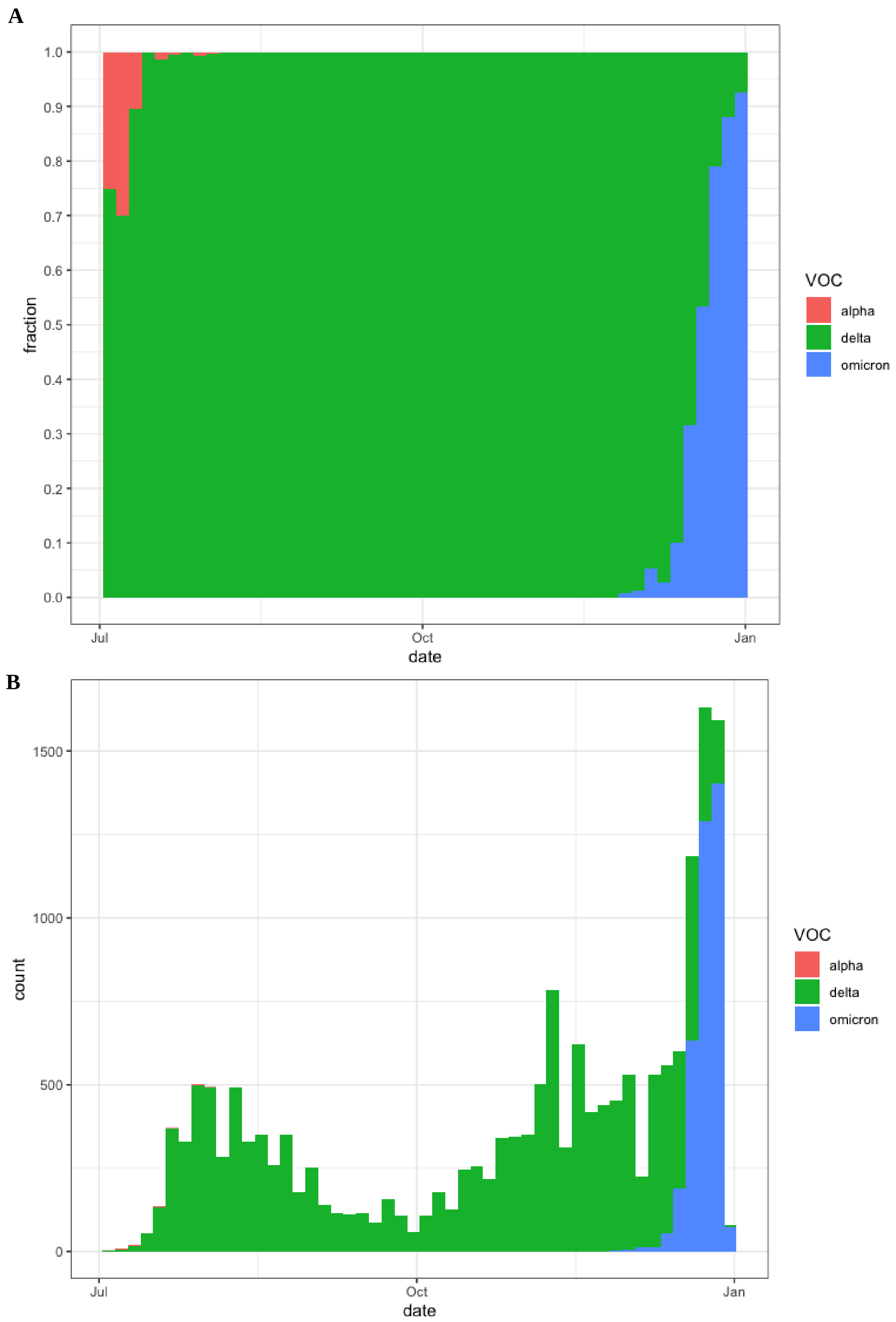

Figure S6 A) Fraction of SARS-CoV-2 infections by variant of concern as determined using sequencing. B) Absolute number of SARS-CoV-2 infections by variant. Shown is the time period July 1, 2021-January 8, 2022.

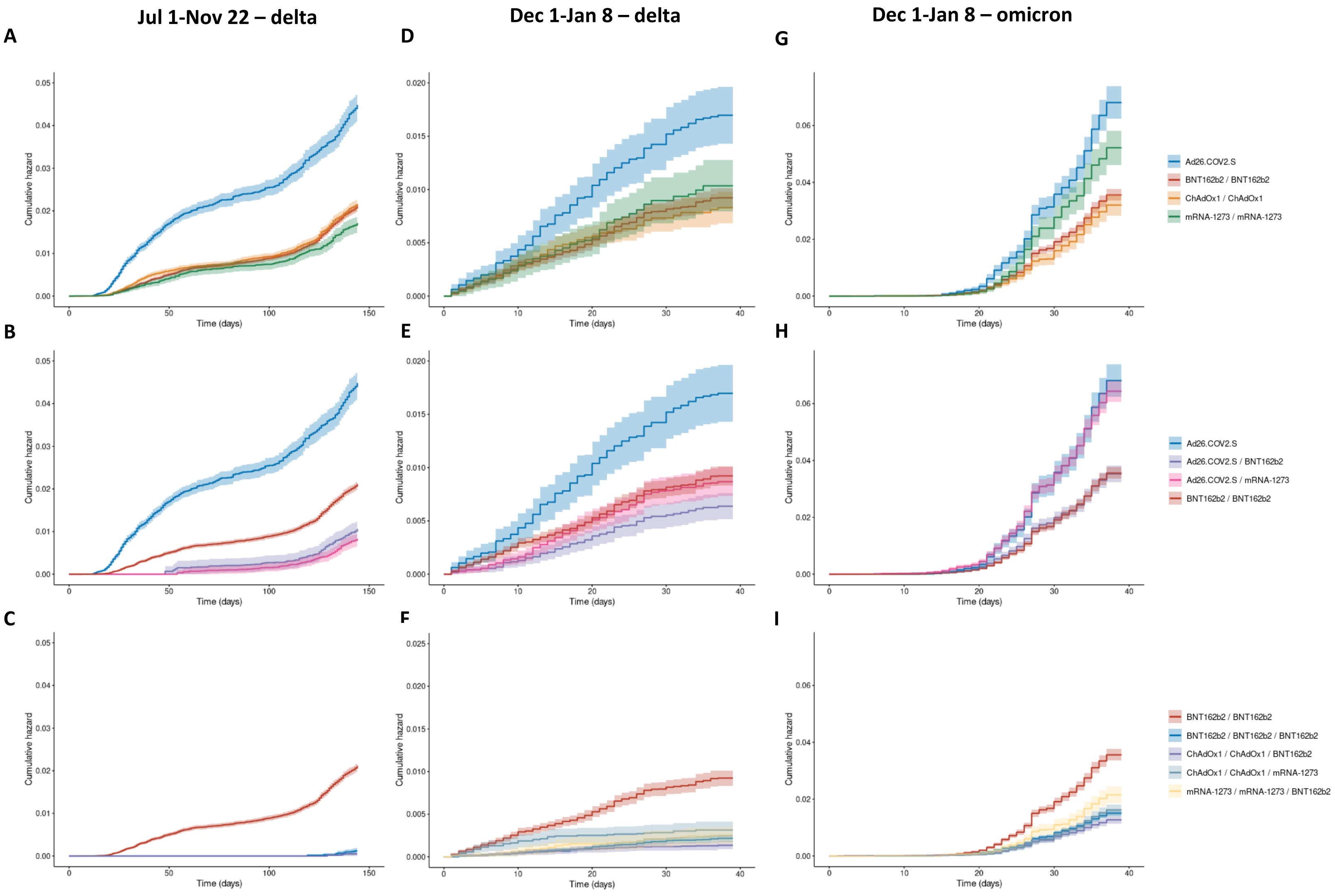

Figure S7. Kaplan-Meier plots comparing SARS-CoV-2 infections. A-C) Infections caused by the delta variant July 1 – November 22, 2021. D-F) Infections caused by the delta variant December 1, 2022– January 8, 2022. G-I) Infections caused by the omicron variant December 1, 2022– January 8, 2022. Top row: Recommended vaccine schedules. Middle row: mRNA booster vaccination of Ad26.COV2.S recipients with 2xBNT162b2 as reference. Bottom row: Three dose vaccinations with more than 5,000 vaccinees, with 2xBNT162b2 as reference. Shaded areas for each survival curve represent the 95% confidence interval of the survival rate.

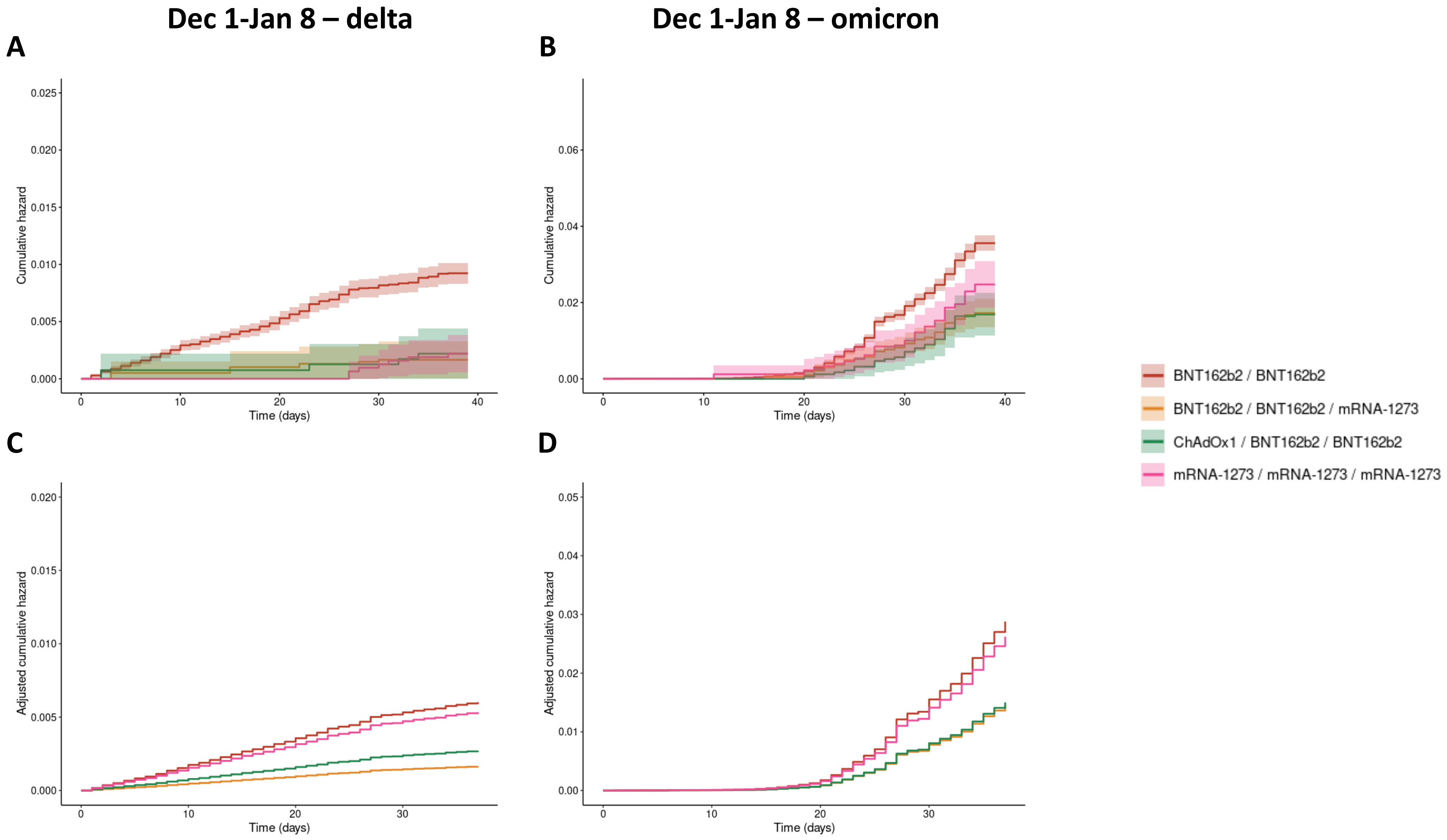

Figure S8. Kaplan-Meier plots comparing SARS-CoV-2 infections, comparison of three dose vaccinations with fewer than 5,000 vaccinees, with 2xBNT162b2 as reference. A and C) Infections caused by the delta variant December 1, 2022– January 8, 2022. B and D) Infections caused by the omicron variant December 1, 2022– January 8, 2022. Top row: Observed cumulative hazard of SARS-CoV-2 infections, shaded areas for each survival curve represent the 95% confidence interval of the survival rate. Bottom row: the adjusted cumulative hazard of SARS-CoV-2 infections.

A

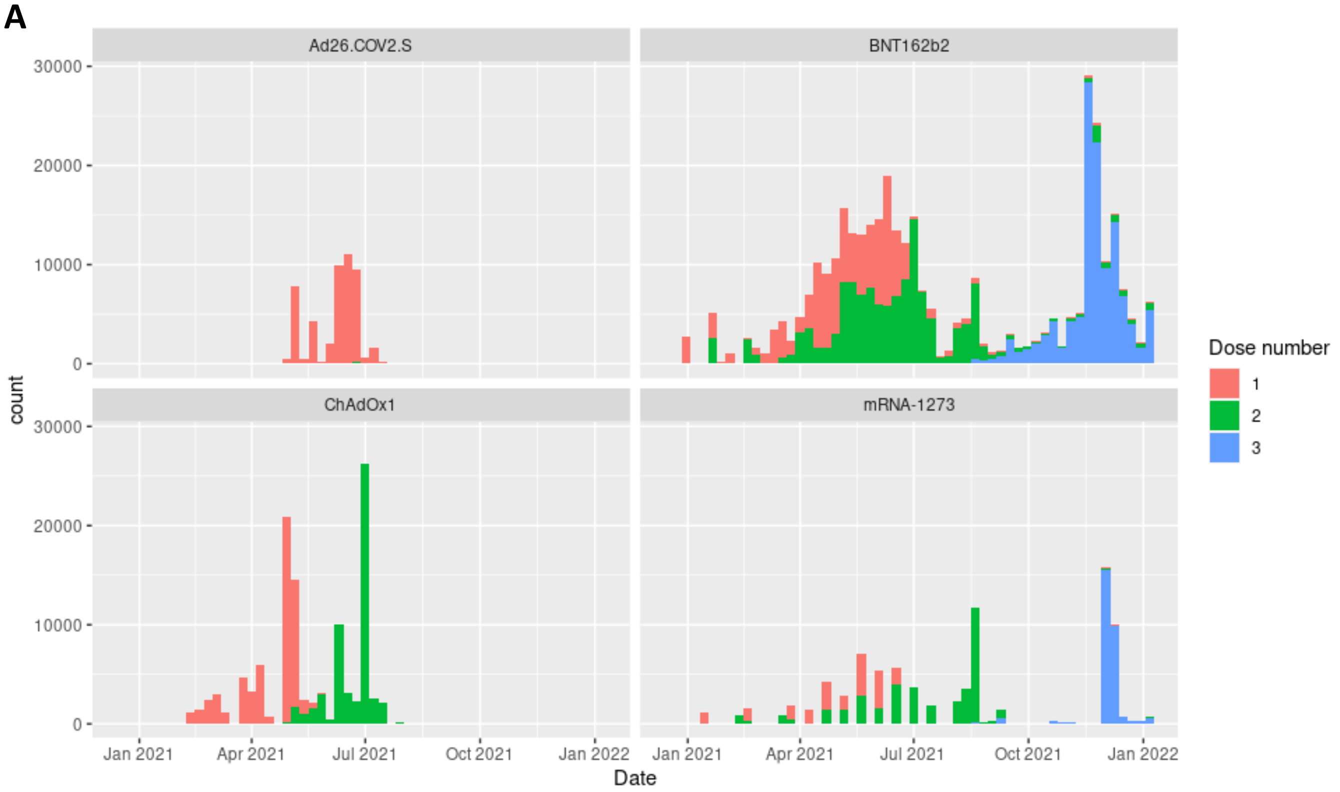

B

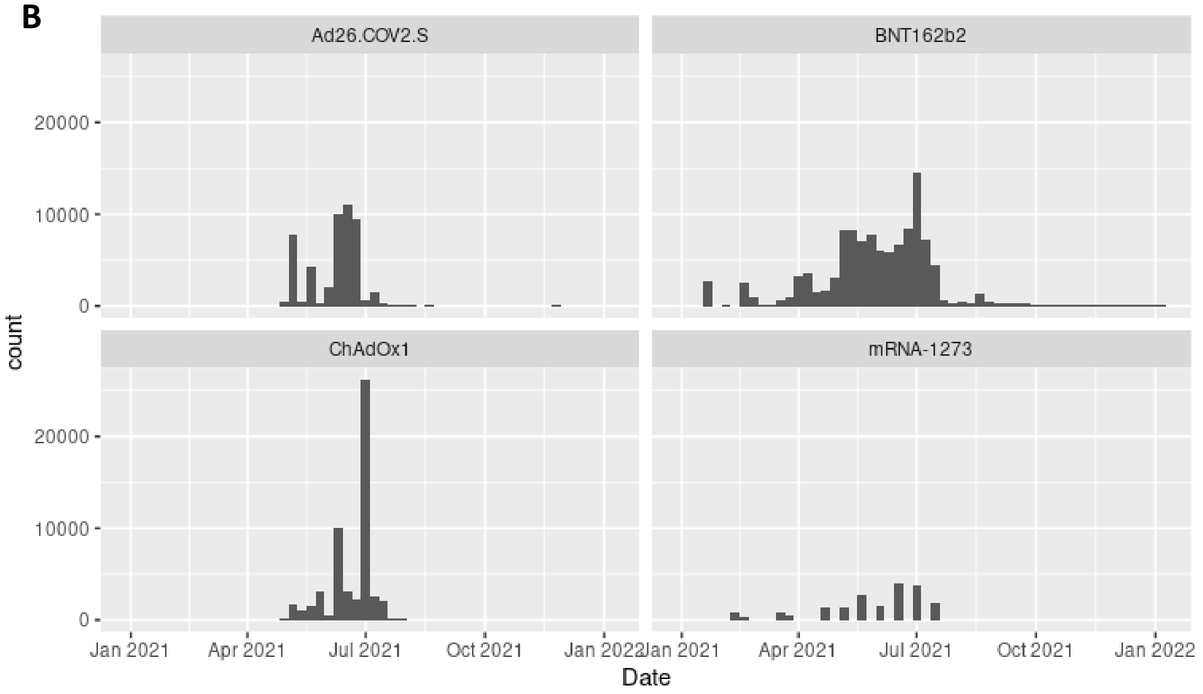

Figure S9. Distribution of when the (a) each dose was administered by vaccine type (b) the final dose of the vaccine according to schedule.

Supplementary tables

Table S1 ACE2-Spike binding inhibition levels relative to the original Spike protein across Spike variants for the different vaccination groups. Vaccine groups within each variant assay were normalized by the median value of the BNT162b2-BNT162b2 reference group. The estimate of the relative effect and confidence intervals are calculated using the normalized value against a null distribution.

Spike variant	BNT162b2 - BNT162b2 reference group (95% CI)	Ad26.COV2.S pre-booster (95% CI)	Ad26.COV2.S – BNT162b2 (95% CI)	Ad26.COV2.S – mRNA-1273 (95% CI)
**Spike**	1.2 (1.0-1.4)	0.23 (0.19-0.28)	0.94 (0.74-1.2)	1.6 (1.3-2.0)
**Spike (B.1.1.7)**	1.1 (0.98-1.3)	0.28 (0.24-0.33)	1.1 (0.86-1.4)	1.8 (1.5-2.2)
**Spike (B.1.351)**	1.1 (0.92-1.2)	0.29 (0.24-0.35)	1.05 (0.83-1.3)	1.6 (1.3-2.0)
**Spike (B.1.526.1)**	1.1 (0.98-1.3)	0.21 (0.17-0.25)	1.04 (0.8-1.3)	1.8 (1.4-2.2)
**Spike (B.1.617.1)**	1.1 (0.93-1.3)	0.19 (0.16-0.23)	0.89 (0.69-1.1)	1.5 (1.2-1.8)
**Spike (B.1.617.2)**	1.1 (0.91-1.3)	0.22 (0.17-0.27)	1.1 (0.83-1.5)	1.7 (1.4-2.2)
**Spike (B.1.617.3)**	1.1 (0.91-1.2)	0.26 (0.21-0.31)	1.0 (0.78-1.3)	1.5 (1.2-1.8)
**Spike (B.1.617)**	1.1 (0.92-1.2)	0.21 (0.17-0.26)	0.87 (0.67-1.1)	1.4 (1.2-1.8)
**Spike (P.1)**	1.1 (0.9-1.2)	0.23 (0.19-0.29)	0.96 (0.73-1.3)	1.6 (1.3-2.0)
**Spike (P.2)**	1.0 (0.88-1.2)	0.21 (0.17-0.25)	0.93 (0.73-1.2)	1.5 (1.2-1.8)

Table S2 Demographics for the ChAdOx1- ChAdOx1-mRNA-1273 recipients.

	**Overall**
Number	17
Female (%)	3 (18%)
Age (mean)	58.4 years
Time from ChAdOx1-ChAdOx1 vaccination	101 days

Table S3 Demographics of vaccination groups on January 8, 2022.

	**January 8 2022**				
**Vaccine**	**Number of persons**	**Age (IQR)**	**Female (%)**	**Days between dose number 2 and 3. Mean (IQR)**	**Days between dose number 1 and 2. Mean (IQR)**
BNT162b2	1,952	39 (27-48)	49.3		
BNT162b2/BNT162b2	28,468	36.5 (25-43)	52.7		25.1 (21-25)
BNT162b2/BNT162b2/BNT162b2	69,206	49.3 (36-64)	54.2	185.7 (167-196)	22.0 (21-22)
BNT162b2/BNT162b2/mRNA-1273	5,892	43.8 (34-52)	62.5	169.4 (154-183)	22.5 (21-22)
mRNA-1273/mRNA-1273	5,131	39.5 (30-47)	52.8		30.8 (26-30)
mRNA-1273/mRNA-1273/mRNA-1273	3,403	50 (43-54)	52.7	171.3 (161-177)	27.7 (26-28)
mRNA-1273/mRNA-1273/BNT162b2	10,482	49 (38-57)	65.8	190.8 (162-196)	28.3 (26-30)
ChAdOx1/ChAdOx1	6,896	52.4 (44-63)	41.0		69.1 (56-77)
ChAdOx1/ChAdOx1/BNT162b2	29,219	61.4 (56-72)	48.2	152.9 (141-169)	67.8 (62-72)
ChAdOx1/ChAdOx1/mRNA-1273	17,148	59.5 (56-64)	36.0	155.5 (152-156)	60.6 (56-63)
ChAdOx1/BNT162b2/BNT162b2	2,263	45.5 (32-58)	70.5	169.3 (154-186)	74.8 (63-87)
ChAdOx1/BNT162b2/mRNA-1273	1,218	56.9 (52-62)	24.9	153.7 (152-152)	60.7 (56-63)
Ad26.COV2.S	8,377	31.8 (24-37)	38.2		
Ad26.COV2.S/BNT162b2	17,670	34.8 (26-43)	39.6		86.8 (59-97)
Ad26.COV2.S/mRNA-1273	18,233	32.5 (24-40)	46.9		73.3 (63-89)

Table S4 Risk of PCR-confirmed COVID-19 diagnosis by vaccination schedules relative to two dose BNT162b2 vaccination for persons who received their second dose from June 1 to July 15. Number of persons is the average size of each group on July 15, except for Ad26.COV2.S with an additional mRNA vaccine where the number of persons is the total over the study period. Hazard ratios shown are calculated using BNT162b2/BNT162b2 as the reference group. Hazard ratios are adjusted for age, sex, and time from vaccination unless specified.

	July 1 2021 – November 22 2021			
**Vaccine**	**Number of persons**	**Number of events**	**HR (95% CI)**	**HR – not adjusted (95% CI)**
BNT162b2/BNT162b2	38,366	1085	1.0 (reference)	1.0 (reference)
mRNA-1273/mRNA-1273	9,140	169	0.7 (0.62-0.86)	0.7 (0.57-0.82)
ChAdOx1/ChAdOx1	41,959	845	1.9 (1.70-2.23)	0.88 (0.8-0.96)
Ad26.COV2.S	30,853	876	2.5 (2.22-2.75)	2.24 (2.15-2.58)
Ad26.COV2.S/BNT162b2	10,120	98	0.7 (0.56-0.86)	0.66 (0.54-0.81)
Ad26.COV2.S/mRNA-1273	12,904	85	0.5 (0.37-0.58)	0.45 (0.36-0.56)

Table S5 Risk of PCR-confirmed COVID-19 diagnosis by vaccination schedules relative to two dose BNT162b2 vaccination from December 1 2021 – January 8 2022 for persons aged 30-50 who received their second dose of BNT162b2 or mRNA-1273 from June 1 2021 – July 15 2021. Hazard ratios shown are calculated using BNT162b2/BNT162b2 as the reference group. Hazard ratios are adjusted for age, sex, and time from vaccination unless specified.

	December 1 2021 – January 8 2022							
**Vaccine**	**Number of persons**	**Number of events (total)**	**Number of events (omicron)**	**HR of omicron (95% CI)**	**HR of omicron – not adjusted (95% CI)**	**Number of events (delta)**	**HR of delta (95% CI)**	**HR of delta – not adjusted (95% CI)**
BNT162b2/BNT162b2	25,162	1,219	573	1.0 (reference)	1.0 (reference)	217	1.0 (reference)	1.0 (reference)
mRNA-1273/mRNA-1273	6,780	409	240	1.5 (1.21-1.84)	1.54 (1.25-1.88)	58	1.1 (0.78-1.5)	0.97 (0.7-1.33)

Table S6 Risk of PCR-confirmed COVID-19 diagnosis by vaccination schedules relative to two dose BNT162b2 vaccination from December 1 2021 – January 8 2022. Number of persons is the average size of each group over the indicated study periods due to vaccinations administered during the study periods. Hazard ratios shown are calculated using BNT162b2/BNT162b2 as the reference group. Hazard ratios are not adjusted for covariates.

	July 1 2021 – November 22 2021			December 1 2021 – January 8 2022					
**Vaccine**	**Number of persons**	**Number of events**	**HR (95% CI)**	**Number of persons**	**Number of events (total)**	**Number of events (Omicron)**	**HR of Omicron (95% CI)**	**Number of events (Delta)**	**HR of Delta (95% CI)**
BNT162b2	3,761	72	1.4 (1.11-1.79)	1,776	123	59	0.94 (0.72-1.22)	30	1.86 (1.28-2.7)
BNT162b2/BNT162b2	96,619	2,022	1.0 (reference)	42,900	2,347	1,175	1.0 (reference)	414	1.0 (reference)
BNT162b2/BNT162b2/BNT162b2	2,796	12	0.12 (0.065-0.20)	56,253	1,856	946	0.42 (0.39-0.46)	119	0.24 (0.19-0.29)
BNT162b2/BNT162b2/mRNA-1273	-		-	3,735	169	90	0.47 (0.38-0.59)	5	0.16 (0.067-0.39)
mRNA-1273	607	7	0.82 (0.39-1.72)	-	-	-	-	-	-
mRNA-1273/mRNA-1273	18,618	319	0.82 (0.72-0.92)	7,167	474	285	1.45 (1.27-1.64)	76	1.11 (0.87-1.41)
mRNA-1273/mRNA-1273/mRNA-1273	-		-	2,023	126	74	0.69 (0.54-0.87)	7	0.43 (0.2-0.9)
mRNA-1273/mRNA-1273/BNT162b2	-		-	9,681	366	215	0.61 (0.52-0.7)	24	0.27 (0.18-0.41)
ChAdOx1	1,418	19	1.09 (0.69-1.71)	-	-	-	-	-	-
ChAdOx1/ChAdOx1	51,556	1,089	1.02 (0.95-1.1)	15,342	585	303	0.88 (0.78-1.0)	145	0.96 (0.79-1.15)
ChAdOx1/BNT162b2	4,192	61	0.68 (0.53-0.88)	-	-	-	-	-	-
ChAdOx1/ChAdOx1/BNT162b2	1682	3	0.045 (0.015-0.14)	23,524	639	341	0.36 (0.32-0.41)	30	0.14 (0.1-0.21)
ChAdOx1/ChAdOx1/mRNA-1273	-		-	14,322	500	265	0.46 (0.4-0.53)	41	0.32 (0.23-0.44)
ChAdOx1/BNT162b2/BNT162b2	-		-	1,747	77	35	0.48 (0.34-0.67)	4	0.26 (0.1-0.7)
ChAdOx1/BNT162b2/mRNA-1273	-		-	1,111	30	20	0.48 (0.31-0.74)	2	0.2 (0.05-0.8)
Ad26.COV2.S	24,959	1,149	2.44 (2.27-2.64)	8,941	1,029	565	1.91 (1.73-2.11)	155	1.85 (1.54-2.22)
Ad26.COV2.S/BNT162b2	9,115	142	0.65 (0.55-0.77)	16,633	1,126	590	0.99 (0.9-1.1)	106	0.69 (0.56-0.86)
Ad26.COV2.S/mRNA-1273	10,789	141	0.55 (0.46-0.65)	17,714	1,801	1,117	1.81 (1.67-1.97)	155	0.94 (0.79-1.14)

Table S7 PCR primers for amplification of viral genomes.

name	pool	seq	length	%gc	tm
**nCoV-2019_1_LEFT**	nCoV-2019_1	ACCAACCAACTTTCGATCTCTTGT	24	41.67	60.69
**nCoV-2019_2_RIGHT**	nCoV-2019_2	TAAGGATCAGTGCCAAGCTCGT	22	50	61.74
**nCoV-2019_3_LEFT**	nCoV-2019_1	CGGTAATAAAGGAGCTGGTGGC	22	54.55	61.32
**nCoV-2019_4_RIGHT**	nCoV-2019_2	CACAAGTAGTGGCACCTTCTTTAGT	25	44	60.97
**nCoV-2019_5_LEFT**	nCoV-2019_1	TGGTGAAACTTCATGGCAGACG	22	50	61.39
**nCoV-2019_6_RIGHT**	nCoV-2019_2	TAGCGGCCTTCTGTAAAACACG	22	50	61.18
**nCoV-2019_7_LEFT**	nCoV-2019_1	ATCAGAGGCTGCTCGTGTTGTA	22	50	61.73
**nCoV-2019_8_RIGHT**	nCoV-2019_2	GCTTCAACAGCTTCACTAGTAGGT	24	45.83	60.56
**nCoV-2019_9_LEFT**	nCoV-2019_1	TCCCACAGAAGTGTTAACAGAGGA	24	45.83	61.18
**nCoV-2019_10_RIGHT**	nCoV-2019_2	TCATCTAACCAATCTTCTTCTTGCTCT	27	37.04	60.31
**nCoV-2019_11_LEFT**	nCoV-2019_1	GGAATTTGGTGCCACTTCTGCT	22	50	61.66
**nCoV-2019_12_RIGHT**	nCoV-2019_2	TTCACTCTTCATTTCCAAAAAGCTTGA	27	33.33	60.36
**nCoV-2019_13_LEFT**	nCoV-2019_1	TCGCACAAATGTCTACTTAGCTGT	24	41.67	60.56
**nCoV-2019_14_RIGHT**	nCoV-2019_2	AGTTTCCACACAGACAGGCATT	22	45.45	60.42
**nCoV-2019_15_LEFT**	nCoV-2019_1	ACAGTGCTTAAAAAGTGTAAAAGTGCC	27	37.04	61.32
**nCoV-2019_16_RIGHT**	nCoV-2019_2	CACAACTTGCGTGTGGAGGTTA	22	50	61.32
**nCoV-2019_17_LEFT**	nCoV-2019_1	CTTCTTTCTTTGAGAGAAGTGAGGACT	27	40.74	60.69
**nCoV-2019_18_RIGHT**	nCoV-2019_2	AGCTTGTTTACCACACGTACAAGG	24	45.83	61.51
**nCoV-2019_19_LEFT**	nCoV-2019_1	GCTGTTATGTACATGGGCACACT	23	47.83	61.18
**nCoV-2019_20_RIGHT**	nCoV-2019_2	ACGTGGCTTTATTAGTTGCATTGTT	25	36	60.28
**nCoV-2019_21_LEFT**	nCoV-2019_1	TGGCTATTGATTATAAACACTACACACCC	29	37.93	61.49
**nCoV-2019_22_RIGHT**	nCoV-2019_2	ACAGTATTCTTTGCTATAGTAGTCGGC	27	40.74	60.73
**nCoV-2019_23_LEFT**	nCoV-2019_1	ACAACTACTAACATAGTTACACGGTGT	27	37.04	60.26
**nCoV-2019_24_RIGHT**	nCoV-2019_2	ACATTCTAACCATAGCTGAAATCGGG	26	42.31	61.19
**nCoV-2019_25_LEFT**	nCoV-2019_1	GCAATTGTTTTTCAGCTATTTTGCAGT	27	33.33	60.73
**nCoV-2019_26_RIGHT**	nCoV-2019_2	TCCGCACTATCACCAACATCAG	22	50	60.42
**nCoV-2019_27_LEFT**	nCoV-2019_1	ACTACAGTCAGCTTATGTGTCAACC	25	44	60.8
**nCoV-2019_28_RIGHT**	nCoV-2019_2	TGTTTAGACATGACATGAACAGGTGT	26	38.46	60.91
**nCoV-2019_29_LEFT**	nCoV-2019_1	ACTTGTGTTCCTTTTTGTTGCTGC	24	41.67	61.39
**nCoV-2019_30_RIGHT**	nCoV-2019_2	ACCACTAGTAGATACACAAACACCAG	26	42.31	60.3
**nCoV-2019_31_LEFT**	nCoV-2019_1	TTCTGAGTACTGTAGGCACGGC	22	54.55	62.03
**nCoV-2019_32_RIGHT**	nCoV-2019_2	AGCACATCACTACGCAACTTTAGA	24	41.67	60.56
**nCoV-2019_33_LEFT**	nCoV-2019_1	ACTTTTGAAGAAGCTGCGCTGT	22	45.45	61.58
**nCoV-2019_34_RIGHT**	nCoV-2019_2	AGTGAAATTGGGCCTCATAGCA	22	45.45	60.03
**nCoV-2019_35_LEFT**	nCoV-2019_1	TGTTCGCATTCAACCAGGACAG	22	50	61.39
**nCoV-2019_36_RIGHT**	nCoV-2019_2	GAACAAAGACCATTGAGTACTCTGGA	26	42.31	60.74
**nCoV-2019_37_LEFT**	nCoV-2019_1	ACACACCACTGGTTGTTACTCAC	23	47.83	60.93
**nCoV-2019_38_RIGHT**	nCoV-2019_2	CACCAAGAGTCAGTCTAAAGTAGCG	25	48	61.13
**nCoV-2019_39_LEFT**	nCoV-2019_1	AGTATTGCCCTATTTTCTTCATAACTGGT	29	34.48	61
**nCoV-2019_40_RIGHT**	nCoV-2019_2	CATGGCTGCATCACGGTCAAAT	22	50	62.09
**nCoV-2019_41_LEFT**	nCoV-2019_1	GTTCCCTTCCATCATATGCAGCT	23	47.83	60.75
**nCoV-2019_42_RIGHT**	nCoV-2019_2	CCTACCTCCCTTTGTTGTGTTGT	23	47.83	60.69
**nCoV-2019_43_LEFT**	nCoV-2019_1	TACGACAGATGTCTTGTGCTGC	22	50	60.93
**nCoV-2019_44_RIGHT**	nCoV-2019_2	AACCTTTCCACATACCGCAGAC	22	50	60.87

**nCoV-2019_45_LEFT**	nCoV-2019_1	TACCTACAACTTGTGCTAATGACCC	25	44	60.57
**nCoV-2019_46_RIGHT**	nCoV-2019_2	CACGTTCACCTAAGTTGGCGTA	22	50	60.86
**nCoV-2019_47_LEFT**	nCoV-2019_1	AGGACTGGTATGATTTTGTAGAAAACCC	28	39.29	61.42
**nCoV-2019_48_RIGHT**	nCoV-2019_2	TAGATTACCAGAAGCAGCGTGC	22	50	60.74
**nCoV-2019_49_LEFT**	nCoV-2019_1	AGGAATTACTTGTGTATGCTGCTGA	25	40	60.57
**nCoV-2019_50_RIGHT**	nCoV-2019_2	TAACATGTTGTGCCAACCACCA	22	45.45	60.95
**nCoV-2019_51_LEFT**	nCoV-2019_1	TCAATAGCCGCCACTAGAGGAG	22	54.55	61.34
**nCoV-2019_52_RIGHT**	nCoV-2019_2	GTTGAGAGCAAAATTCATGAGGTCC	25	44	60.62
**nCoV-2019_53_LEFT**	nCoV-2019_1	AGCAAAATGTTGGACTGAGACTGA	24	41.67	60.69
**nCoV-2019_54_RIGHT**	nCoV-2019_2	AACCAAAAACTTGTCCATTAGCACA	25	36	60.11
**nCoV-2019_55_LEFT**	nCoV-2019_1	ACTCAACTTTACTTAGGAGGTATGAGCT	28	39.29	61.43
**nCoV-2019_56_RIGHT**	nCoV-2019_2	ACACTATGCGAGCAGAAGGGTA	22	50	61.21
**nCoV-2019_57_LEFT**	nCoV-2019_1	ATTCTACACTCCAGGGACCACC	22	54.55	61.16
**nCoV-2019_58_RIGHT**	nCoV-2019_2	CTTTTCTCCAAGCAGGGTTACGT	23	47.83	61.06
**nCoV-2019_59_LEFT**	nCoV-2019_1	TCACGCATGATGTTTCATCTGCA	23	43.48	61.42
**nCoV-2019_60_RIGHT**	nCoV-2019_2	GGTACCAACAGCTTCTCTAGTAGC	24	50	60.44
**nCoV-2019_61_LEFT**	nCoV-2019_1	TGTTTATCACCCGCGAAGAAGC	22	50	61.5
**nCoV-2019_62_RIGHT**	nCoV-2019_2	GTTGAACCTTTCTACAAGCCGC	22	50	60.35
**nCoV-2019_63_LEFT**	nCoV-2019_1	TGTTAAGCGTGTTGACTGGACT	22	45.45	60.16
**nCoV-2019_64_RIGHT**	nCoV-2019_2	AGTCTTGTAAAAGTGTTCCAGAGGT	25	40	60.1
**nCoV-2019_65_LEFT**	nCoV-2019_1	GCTGGCTTTAGCTTGTGGGTTT	22	50	61.92
**nCoV-2019_66_RIGHT**	nCoV-2019_2	TCAATTTCCATTTGACTCCTGGGT	24	41.67	60.45
**nCoV-2019_67_LEFT**	nCoV-2019_1	GTTGTCCAACAATTACCTGAAACTTACT	28	35.71	60.43
**nCoV-2019_68_RIGHT**	nCoV-2019_2	CTCCTTTATCAGAACCAGCACCA	23	47.83	60.31
**nCoV-2019_69_LEFT**	nCoV-2019_1	TGTCGCAAAATATACTCAACTGTGTCA	27	37.04	61.43
**nCoV-2019_70_RIGHT**	nCoV-2019_2	TGACCTTCTTTTAAAGACATAACAGCAG	28	35.71	60.27
**nCoV-2019_71_LEFT**	nCoV-2019_1	ACAAATCCAATTCAGTTGTCTTCCTATTC	29	34.48	60.54
**nCoV-2019_72_RIGHT**	nCoV-2019_2	ACTCTGAACTCACTTTCCATCCAAC	25	44	60.97
**nCoV-2019_73_LEFT**	nCoV-2019_1	CAATTTTGTAATGATCCATTTTTGGGTGT	29	31.03	60.29
**nCoV-2019_74_RIGHT**	nCoV-2019_2	GCAACACAGTTGCTGATTCTCTTC	24	45.83	60.85
**nCoV-2019_75_LEFT**	nCoV-2019_1	AGAGTCCAACCAACAGAATCTATTGT	26	38.46	60.24
**nCoV-2019_76_RIGHT**	nCoV-2019_2	ACACCTGTGCCTGTTAAACCAT	22	45.45	60.42
**nCoV-2019_77_LEFT**	nCoV-2019_1	CCAGCAACTGTTTGTGGACCTA	22	50	60.75
**nCoV-2019_78_RIGHT**	nCoV-2019_2	TGTGTACAAAAACTGCCATATTGCA	25	36	60.22
**nCoV-2019_79_LEFT**	nCoV-2019_1	GTGGTGATTCAACTGAATGCAGC	23	47.83	60.92
**nCoV-2019_80_RIGHT**	nCoV-2019_2	TGGAGCTAAGTTGTTTAACAAGCG	24	41.67	60.02
**nCoV-2019_81_LEFT**	nCoV-2019_1	GCACTTGGAAAACTTCAAGATGTGG	25	44	61.24
**nCoV-2019_82_RIGHT**	nCoV-2019_2	TGCCAGAGATGTCACCTAAATCAA	24	41.67	60.02
**nCoV-2019_83_LEFT**	nCoV-2019_1	TCCTTTGCAACCTGAATTAGACTCA	25	40	60.46
**nCoV-2019_84_RIGHT**	nCoV-2019_2	AGGTGTGAGTAAACTGTTACAAACAAC	27	37.04	60.36
**nCoV-2019_85_LEFT**	nCoV-2019_1	ACTAGCACTCTCCAAGGGTGTT	22	50	61.03
**nCoV-2019_86_RIGHT**	nCoV-2019_2	ACGAAAGCAAGAAAAAGAAGTACGC	25	40	61.01
**nCoV-2019_87_LEFT**	nCoV-2019_1	CGACTACTAGCGTGCCTTTGTA	22	50	60.16
**nCoV-2019_88_RIGHT**	nCoV-2019_2	TGGTCAGAATAGTGCCATGGAGT	23	47.83	61.4
**nCoV-2019_89_LEFT**	nCoV-2019_1	GTACGCGTTCCATGTGGTCATT	22	50	61.5
**nCoV-2019_90_RIGHT**	nCoV-2019_2	TGAAATGGTGAATTGCCCTCGT	22	45.45	60.82
**nCoV-2019_91_LEFT**	nCoV-2019_1	TCACTACCAAGAGTGTGTTAGAGGT	25	44	60.93
**nCoV-2019_92_RIGHT**	nCoV-2019_2	AGGTTCCTGGCAATTAATTGTAAAAGG	27	37.04	60.53
**nCoV-2019_93_LEFT**	nCoV-2019_1	TGAGGCTGGTTCTAAATCACCCA	23	47.83	61.59
**nCoV-2019_94_RIGHT**	nCoV-2019_2	TTTGGCAATGTTGTTCCTTGAGG	23	43.48	60.18
**nCoV-2019_95_LEFT**	nCoV-2019_1	TGAGGGAGCCTTGAATACACCA	22	50	61.1
**nCoV-2019_96_RIGHT**	nCoV-2019_2	TAGGCTCTGTTGGTGGGAATGT	22	50	61.36

**nCoV-2019_97_LEFT**	nCoV-2019_1	TGGATGACAAAGATCCAAATTTCAAAGA	28	32.14	60.22
**nCoV-2019_98_RIGHT**	nCoV-2019_2	TTCTCCTAAGAAGCTATTAAAATCACATGG	30	33.33	60.01

Supplementary references

1. Hjorleifsson KE, Rognvaldsson S, Jonsson H, et al. Reconstruction of a large-scale outbreak of SARS-CoV-2 infection in Iceland informs vaccination strategies. medRxiv 2021;2021.06.11.21258741.

2. Gudbjartsson DF, Helgason A, Jonsson H, et al. Spread of SARS-CoV-2 in the Icelandic Population. N Engl J Med 2020;382(24):2302–15.
